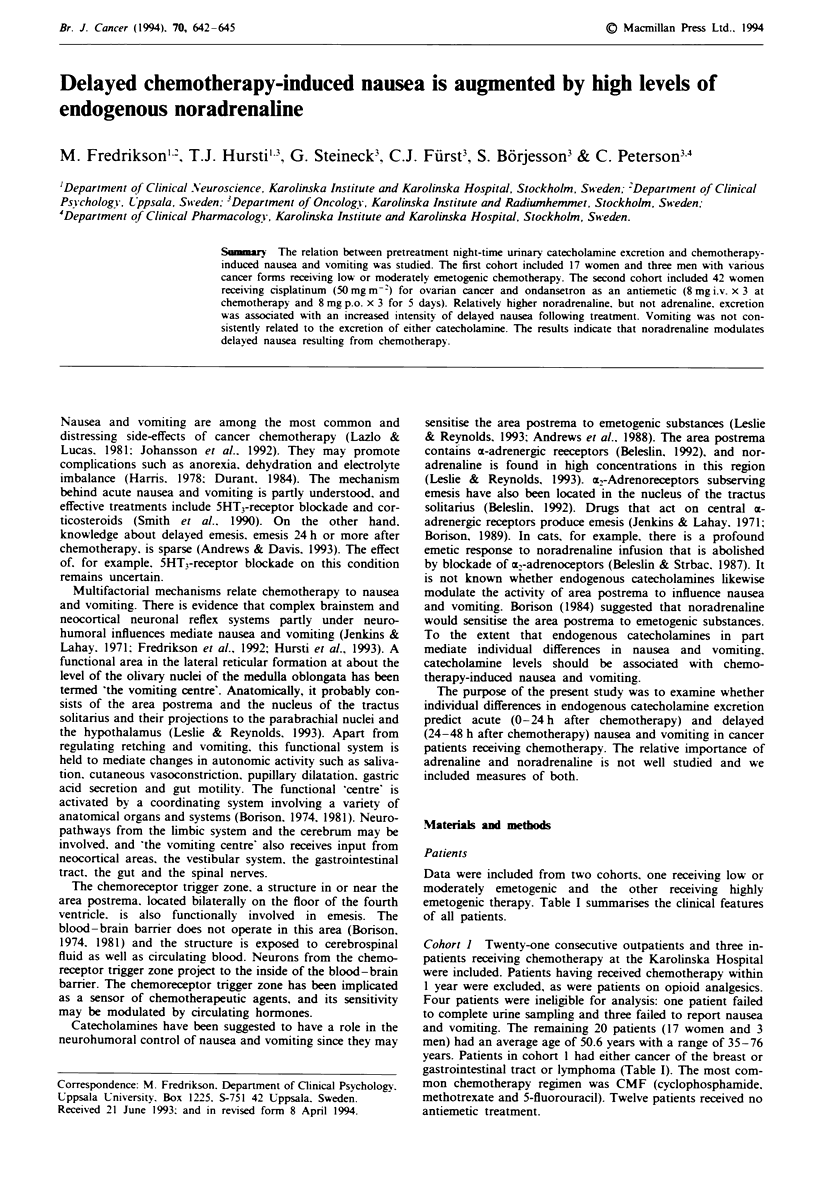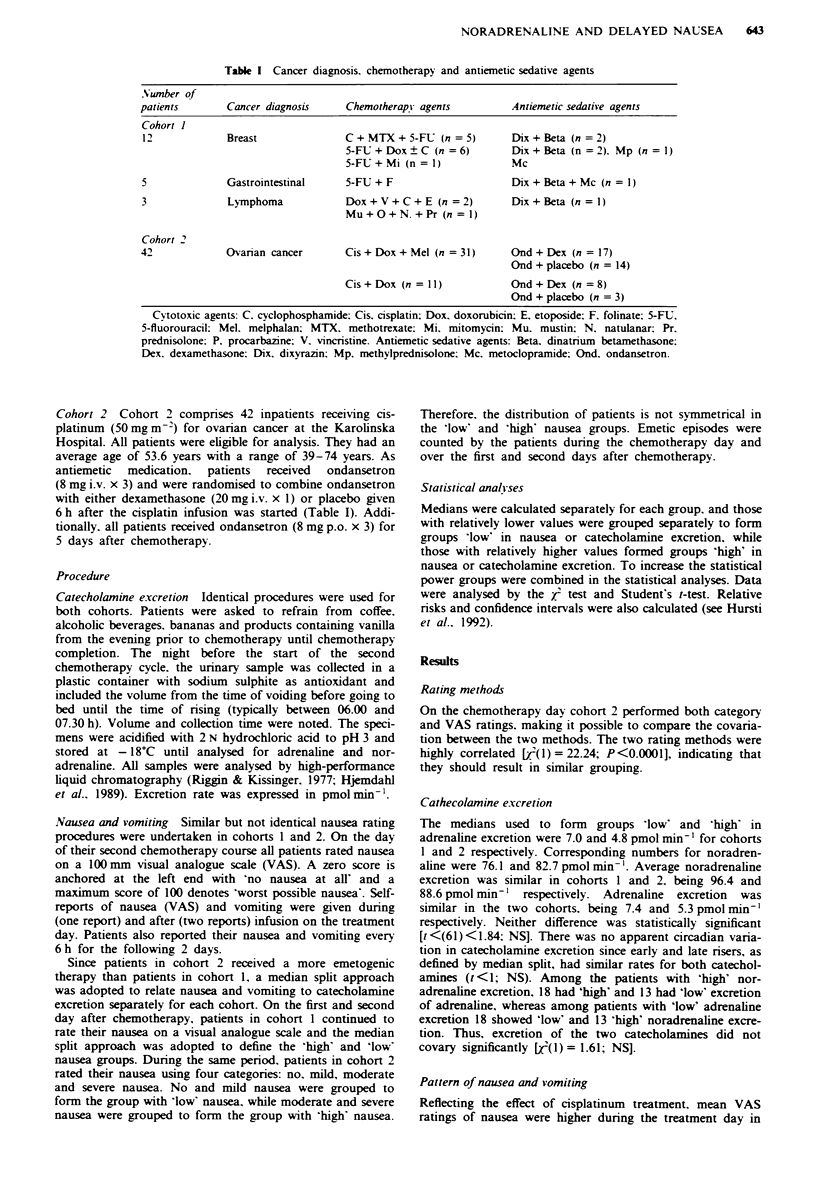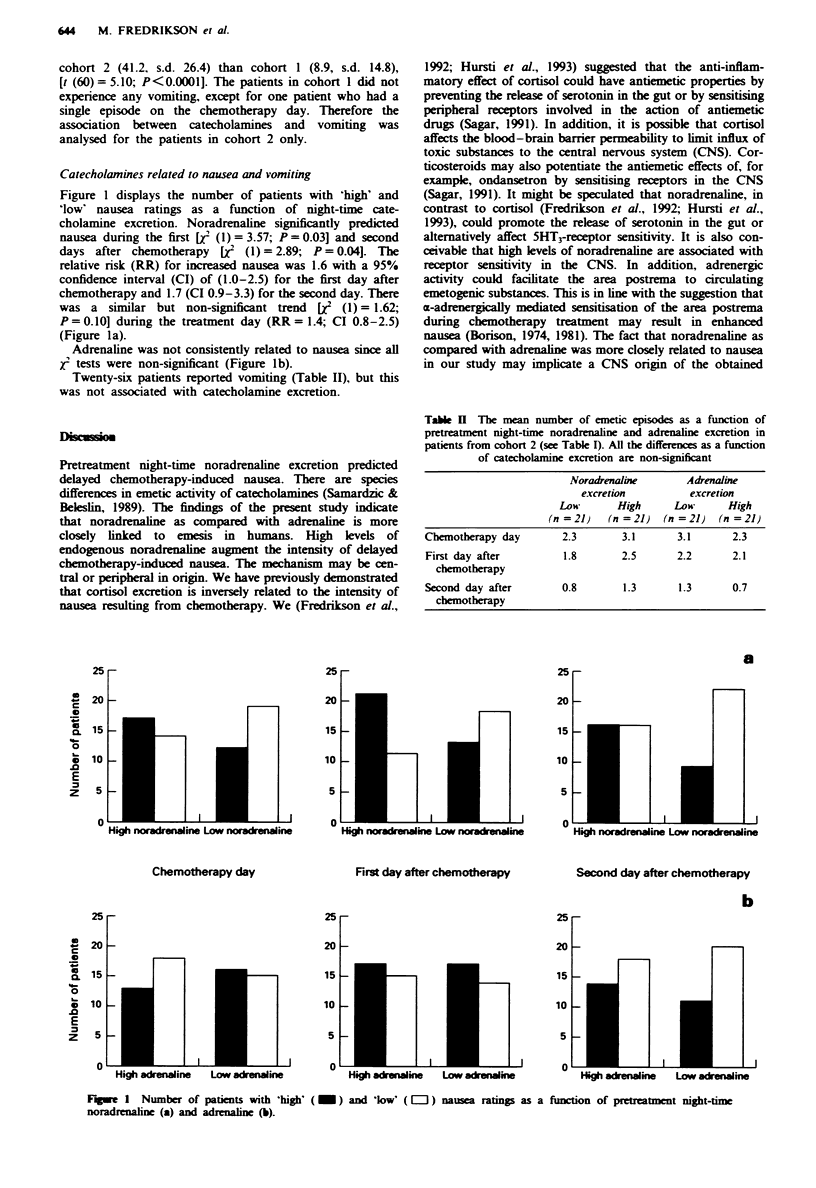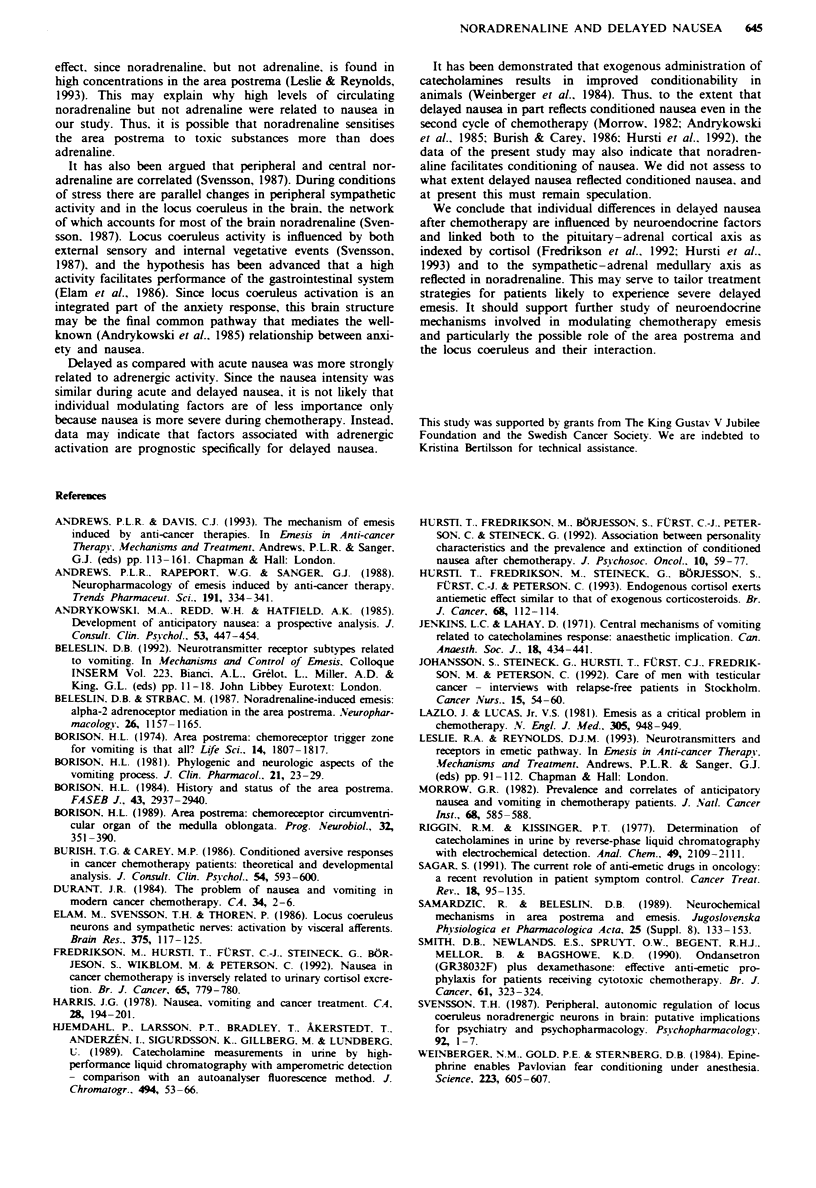# Delayed chemotherapy-induced nausea is augmented by high levels of endogenous noradrenaline.

**DOI:** 10.1038/bjc.1994.364

**Published:** 1994-10

**Authors:** M. Fredrikson, T. J. Hursti, G. Steineck, C. J. Fürst, S. Börjesson, C. Peterson

**Affiliations:** Department of Clinical Neuroscience, Karolinska Institute, Stockholm, Sweden.

## Abstract

The relation between pretreatment night-time urinary catecholamine excretion and chemotherapy-induced nausea and vomiting was studied. The first cohort included 17 women and three men with various cancer forms receiving low or moderately emetogenic chemotherapy. The second cohort included 42 women receiving cisplatinum (50 mg m-2) for ovarian cancer and ondansetron as an antiemetic (8 mg i.v. x 3 at chemotherapy and 8 mg p.o. x 3 for 5 days). Relatively higher noradrenaline, but not adrenaline, excretion was associated with an increased intensity of delayed nausea following treatment. Vomiting was not consistently related to the excretion of either catecholamine. The results indicate that noradrenaline modulates delayed nausea resulting from chemotherapy.


Br. J. Cancer (1994), 70, 642-645 © Macmillan Press Ltd., 1994

### Delayed chemotherapy-induced nausea is augmented by high levels of endogenous noradrenaline

M. Fredrikson[1,2], T.J. Hursti[1,3], G. Steineck[3], C.J. Fürst[3], S. Börjesson[3] & C. Peterson[3,4]

[1]Department of Clinical Neuroscience, Karolinska Institute and Karolinska Hospital, Stockholm, Sweden; [2]Department of Clinical Psychology, Uppsala, Sweden; [3]Department of Oncology, Karolinska Institute and Radiumhemmet, Stockholm, Sweden; [4]Department of Clinical Pharmacology, Karolinska Institute and Karolinska Hospital, Stockholm, Sweden.

**Summary** The relation between pretreatment night-time urinary catecholamine excretion and chemotherapy-induced nausea and vomiting was studied. The first cohort included 17 women and three men with various cancer forms receiving low or moderately emetogenic chemotherapy. The second cohort included 42 women receiving cisplatinum (50 mg $m^{-2}$) for ovarian cancer and ondansetron as an antiemetic (8 mg i.v. × 3 at chemotherapy and 8 mg p.o. × 3 for 5 days). Relatively higher noradrenaline, but not adrenaline, excretion was associated with an increased intensity of delayed nausea following treatment. Vomiting was not consistently related to the excretion of either catecholamine. The results indicate that noradrenaline modulates delayed nausea resulting from chemotherapy.

Nausea and vomiting are among the most common and distressing side-effects of cancer chemotherapy (Lazlo & Lucas, 1981; Johansson *et al.*, 1992). They may promote complications such as anorexia, dehydration and electrolyte imbalance (Harris, 1978; Durant, 1984). The mechanism behind acute nausea and vomiting is partly understood, and effective treatments include $5HT_3$-receptor blockade and corticosteroids (Smith *et al.*, 1990). On the other hand, knowledge about delayed emesis, emesis 24 h or more after chemotherapy, is sparse (Andrews & Davis, 1993). The effect of, for example, $5HT_3$-receptor blockade on this condition remains uncertain.

Multifactorial mechanisms relate chemotherapy to nausea and vomiting. There is evidence that complex brainstem and neocortical neuronal reflex systems partly under neurohumoral influences mediate nausea and vomiting (Jenkins & Lahay, 1971; Fredrikson *et al.*, 1992; Hursti *et al.*, 1993). A functional area in the lateral reticular formation at about the level of the olivary nuclei of the medulla oblongata has been termed 'the vomiting centre'. Anatomically, it probably consists of the area postrema and the nucleus of the tractus solitarius and their projections to the parabrachial nuclei and the hypothalamus (Leslie & Reynolds, 1993). Apart from regulating retching and vomiting, this functional system is held to mediate changes in autonomic activity such as salivation, cutaneous vasoconstriction, pupillary dilatation, gastric acid secretion and gut motility. The functional 'centre' is activated by a coordinating system involving a variety of anatomical organs and systems (Borison, 1974, 1981). Neuropathways from the limbic system and the cerebrum may be involved, and 'the vomiting centre' also receives input from neocortical areas, the vestibular system, the gastrointestinal tract, the gut and the spinal nerves.

The chemoreceptor trigger zone, a structure in or near the area postrema, located bilaterally on the floor of the fourth ventricle, is also functionally involved in emesis. The blood-brain barrier does not operate in this area (Borison, 1974, 1981) and the structure is exposed to cerebrospinal fluid as well as circulating blood. Neurons from the chemoreceptor trigger zone project to the inside of the blood-brain barrier. The chemoreceptor trigger zone has been implicated as a sensor of chemotherapeutic agents, and its sensitivity may be modulated by circulating hormones.

Catecholamines have been suggested to have a role in the neurohumoral control of nausea and vomiting since they may sensitise the area postrema to emetogenic substances (Leslie & Reynolds, 1993; Andrews *et al.*, 1988). The area postrema contains α-adrenergic reeceptors (Beleslin, 1992), and noradrenaline is found in high concentrations in this region (Leslie & Reynolds, 1993). $\alpha_2$-Adrenoreceptors subserving emesis have also been located in the nucleus of the tractus solitarius (Beleslin, 1992). Drugs that act on central α-adrenergic receptors produce emesis (Jenkins & Lahay, 1971; Borison, 1989). In cats, for example, there is a profound emetic response to noradrenaline infusion that is abolished by blockade of $\alpha_2$-adrenoceptors (Beleslin & Strbac, 1987). It is not known whether endogenous catecholamines likewise modulate the activity of area postrema to influence nausea and vomiting. Borison (1984) suggested that noradrenaline would sensitise the area postrema to emetogenic substances. To the extent that endogenous catecholamines in part mediate individual differences in nausea and vomiting, catecholamine levels should be associated with chemotherapy-induced nausea and vomiting.

The purpose of the present study was to examine whether individual differences in endogenous catecholamine excretion predict acute (0-24 h after chemotherapy) and delayed (24-48 h after chemotherapy) nausea and vomiting in cancer patients receiving chemotherapy. The relative importance of adrenaline and noradrenaline is not well studied and we included measures of both.

### Materials and methods

### *Patients*

Data were included from two cohorts, one receiving low or moderately emetogenic and the other receiving highly emetogenic therapy. Table I summarises the clinical features of all patients.

*Cohort 1* Twenty-one consecutive outpatients and three inpatients receiving chemotherapy at the Karolinska Hospital were included. Patients having received chemotherapy within 1 year were excluded, as were patients on opioid analgesics. Four patients were ineligible for analysis: one patient failed to complete urine sampling and three failed to report nausea and vomiting. The remaining 20 patients (17 women and 3 men) had an average age of 50.6 years with a range of 35-76 years. Patients in cohort 1 had either cancer of the breast or gastrointestinal tract or lymphoma (Table I). The most common chemotherapy regimen was CMF (cyclophosphamide, methotrexate and 5-fluorouracil). Twelve patients received no antiemetic treatment.

Correspondence: M. Fredrikson, Department of Clinical Psychology, Uppsala University, Box 1225, S-751 42 Uppsala, Sweden.
Received 21 June 1993; and in revised form 8 April 1994.

**Table I** Cancer diagnosis, chemotherapy and antiemetic sedative agents

| Number of patients | Cancer diagnosis | Chemotherapy agents | Antiemetic sedative agents |
|---|---|---|---|
| *Cohort 1* | | | |
| 12 | Breast | C + MTX + 5-FU ($n$ = 5) | Dix + Beta ($n$ = 2) |
| | | 5-FU + Dox ± C ($n$ = 6) | Dix + Beta (n = 2), Mp ($n$ = 1) |
| | | 5-FU + Mi (n = 1) | Mc |
| 5 | Gastrointestinal | 5-FU + F | Dix + Beta + Mc ($n$ = 1) |
| 3 | Lymphoma | Dox + V + C + E ($n$ = 2) | Dix + Beta ($n$ = 1) |
| | | Mu + O + N. + Pr ($n$ = 1) | |
| *Cohort 2* | | | |
| 42 | Ovarian cancer | Cis + Dox + Mel ($n$ = 31) | Ond + Dex ($n$ = 17) |
| | | | Ond + placebo ($n$ = 14) |
| | | Cis + Dox ($n$ = 11) | Ond + Dex ($n$ = 8) |
| | | | Ond + placebo ($n$ = 3) |

Cytotoxic agents: C, cyclophosphamide; Cis, cisplatin; Dox, doxorubicin; E, etoposide; F, folinate; 5-FU, 5-fluorouracil; Mel, melphalan; MTX, methotrexate; Mi, mitomycin; Mu, mustin; N, natulanar; Pr, prednisolone; P, procarbazine; V, vincristine. Antiemetic sedative agents: Beta, dinatrium betamethasone; Dex, dexamethasone; Dix, dixyrazin; Mp, methylprednisolone; Mc, metoclopramide; Ond, ondansetron.

*Cohort 2* Cohort 2 comprises 42 inpatients receiving cisplatinum (50 mg $m^{-2}$) for ovarian cancer at the Karolinska Hospital. All patients were eligible for analysis. They had an average age of 53.6 years with a range of 39–74 years. As antiemetic medication, patients received ondansetron (8 mg i.v. × 3) and were randomised to combine ondansetron with either dexamethasone (20 mg i.v. × 1) or placebo given 6 h after the cisplatin infusion was started (Table I). Additionally, all patients received ondansetron (8 mg p.o. × 3) for 5 days after chemotherapy.

### Procedure

*Catecholamine excretion* Identical procedures were used for both cohorts. Patients were asked to refrain from coffee, alcoholic beverages, bananas and products containing vanilla from the evening prior to chemotherapy until chemotherapy completion. The night before the start of the second chemotherapy cycle, the urinary sample was collected in a plastic container with sodium sulphite as antioxidant and included the volume from the time of voiding before going to bed until the time of rising (typically between 06.00 and 07.30 h). Volume and collection time were noted. The specimens were acidified with 2 N hydrochloric acid to pH 3 and stored at −18°C until analysed for adrenaline and noradrenaline. All samples were analysed by high-performance liquid chromatography (Riggin & Kissinger, 1977; Hjemdahl *et al.*, 1989). Excretion rate was expressed in pmol $min^{-1}$.

*Nausea and vomiting* Similar but not identical nausea rating procedures were undertaken in cohorts 1 and 2. On the day of their second chemotherapy course all patients rated nausea on a 100 mm visual analogue scale (VAS). A zero score is anchored at the left end with 'no nausea at all' and a maximum score of 100 denotes 'worst possible nausea'. Self-reports of nausea (VAS) and vomiting were given during (one report) and after (two reports) infusion on the treatment day. Patients also reported their nausea and vomiting every 6 h for the following 2 days.

Since patients in cohort 2 received a more emetogenic therapy than patients in cohort 1, a median split approach was adopted to relate nausea and vomiting to catecholamine excretion separately for each cohort. On the first and second day after chemotherapy, patients in cohort 1 continued to rate their nausea on a visual analogue scale and the median split approach was adopted to define the 'high' and 'low' nausea groups. During the same period, patients in cohort 2 rated their nausea using four categories: no, mild, moderate and severe nausea. No and mild nausea were grouped to form the group with 'low' nausea, while moderate and severe nausea were grouped to form the group with 'high' nausea. Therefore, the distribution of patients is not symmetrical in the 'low' and 'high' nausea groups. Emetic episodes were counted by the patients during the chemotherapy day and over the first and second days after chemotherapy.

### Statistical analyses

Medians were calculated separately for each group, and those with relatively lower values were grouped separately to form groups 'low' in nausea or catecholamine excretion, while those with relatively higher values formed groups 'high' in nausea or catecholamine excretion. To increase the statistical power groups were combined in the statistical analyses. Data were analysed by the $\chi^2$ test and Student's $t$-test. Relative risks and confidence intervals were also calculated (see Hursti *et al.*, 1992).

### Results

### Rating methods

On the chemotherapy day cohort 2 performed both category and VAS ratings, making it possible to compare the covariation between the two methods. The two rating methods were highly correlated [$\chi^2(1) = 22.24$; $P<0.0001$], indicating that they should result in similar grouping.

### Cathecolamine excretion

The medians used to form groups 'low' and 'high' in adrenaline excretion were 7.0 and 4.8 pmol $min^{-1}$ for cohorts 1 and 2 respectively. Corresponding numbers for noradrenaline were 76.1 and 82.7 pmol $min^{-1}$. Average noradrenaline excretion was similar in cohorts 1 and 2, being 96.4 and 88.6 pmol $min^{-1}$ respectively. Adrenaline excretion was similar in the two cohorts, being 7.4 and 5.3 pmol $min^{-1}$ respectively. Neither difference was statistically significant [$t<(61)<1.84$; NS]. There was no apparent circadian variation in catecholamine excretion since early and late risers, as defined by median split, had similar rates for both catecholamines ($t<1$; NS). Among the patients with 'high' noradrenaline excretion, 18 had 'high' and 13 had 'low' excretion of adrenaline, whereas among patients with 'low' adrenaline excretion 18 showed 'low' and 13 'high' noradrenaline excretion. Thus, excretion of the two catecholamines did not covary significantly [$\chi^2(1) = 1.61$; NS].

### Pattern of nausea and vomiting

Reflecting the effect of cisplatinum treatment, mean VAS ratings of nausea were higher during the treatment day in cohort 2 (41.2, s.d. 26.4) than cohort 1 (8.9, s.d. 14.8), [$t$ (60) = 5.10; $P$<0.0001]. The patients in cohort 1 did not experience any vomiting, except for one patient who had a single episode on the chemotherapy day. Therefore the association between catecholamines and vomiting was analysed for the patients in cohort 2 only.

*Catecholamines related to nausea and vomiting*

Figure 1 displays the number of patients with 'high' and 'low' nausea ratings as a function of night-time catecholamine excretion. Noradrenaline significantly predicted nausea during the first [$\chi^2$ (1) = 3.57; $P$ = 0.03] and second days after chemotherapy [$\chi^2$ (1) = 2.89; $P$ = 0.04]. The relative risk (RR) for increased nausea was 1.6 with a 95% confidence interval (CI) of (1.0–2.5) for the first day after chemotherapy and 1.7 (CI 0.9–3.3) for the second day. There was a similar but non-significant trend [$\chi^2$ (1) = 1.62; $P$ = 0.10] during the treatment day (RR = 1.4; CI 0.8–2.5) (Figure 1a).

Adrenaline was not consistently related to nausea since all $\chi^2$ tests were non-significant (Figure 1b).

Twenty-six patients reported vomiting (Table II), but this was not associated with catecholamine excretion.

### Discussion

Pretreatment night-time noradrenaline excretion predicted delayed chemotherapy-induced nausea. There are species differences in emetic activity of catecholamines (Samardzic & Beleslin, 1989). The findings of the present study indicate that noradrenaline as compared with adrenaline is more closely linked to emesis in humans. High levels of endogenous noradrenaline augment the intensity of delayed chemotherapy-induced nausea. The mechanism may be central or peripheral in origin. We have previously demonstrated that cortisol excretion is inversely related to the intensity of nausea resulting from chemotherapy. We (Fredrikson et al., 1992; Hursti et al., 1993) suggested that the anti-inflammatory effect of cortisol could have antiemetic properties by preventing the release of serotonin in the gut or by sensitising peripheral receptors involved in the action of antiemetic drugs (Sagar, 1991). In addition, it is possible that cortisol affects the blood–brain barrier permeability to limit influx of toxic substances to the central nervous system (CNS). Corticosteroids may also potentiate the antiemetic effects of, for example, ondansetron by sensitising receptors in the CNS (Sagar, 1991). It might be speculated that noradrenaline, in contrast to cortisol (Fredrikson et al., 1992; Hursti et al., 1993), could promote the release of serotonin in the gut or alternatively affect $5HT_3$-receptor sensitivity. It is also conceivable that high levels of noradrenaline are associated with receptor sensitivity in the CNS. In addition, adrenergic activity could facilitate the area postrema to circulating emetogenic substances. This is in line with the suggestion that α-adrenergically mediated sensitisation of the area postrema during chemotherapy treatment may result in enhanced nausea (Borison, 1974, 1981). The fact that noradrenaline as compared with adrenaline was more closely related to nausea in our study may implicate a CNS origin of the obtained

**Table II** The mean number of emetic episodes as a function of pretreatment night-time noradrenaline and adrenaline excretion in patients from cohort 2 (see Table I). All the differences as a function of catecholamine excretion are non-significant

| | *Noradrenaline excretion* | | *Adrenaline excretion* | |
|---|---|---|---|---|
| | *Low (n = 21)* | *High (n = 21)* | *Low (n = 21)* | *High (n = 21)* |
| Chemotherapy day | 2.3 | 3.1 | 3.1 | 2.3 |
| First day after chemotherapy | 1.8 | 2.5 | 2.2 | 2.1 |
| Second day after chemotherapy | 0.8 | 1.3 | 1.3 | 0.7 |

**Figure 1** Number of patients with 'high' (■) and 'low' (□) nausea ratings as a function of pretreatment night-time noradrenaline (**a**) and adrenaline (**b**).

effect, since noradrenaline, but not adrenaline, is found in high concentrations in the area postrema (Leslie & Reynolds, 1993). This may explain why high levels of circulating noradrenaline but not adrenaline were related to nausea in our study. Thus, it is possible that noradrenaline sensitises the area postrema to toxic substances more than does adrenaline.

It has also been argued that peripheral and central noradrenaline are correlated (Svensson, 1987). During conditions of stress there are parallel changes in peripheral sympathetic activity and in the locus coeruleus in the brain, the network of which accounts for most of the brain noradrenaline (Svensson, 1987). Locus coeruleus activity is influenced by both external sensory and internal vegetative events (Svensson, 1987), and the hypothesis has been advanced that a high activity facilitates performance of the gastrointestinal system (Elam *et al.*, 1986). Since locus coeruleus activation is an integrated part of the anxiety response, this brain structure may be the final common pathway that mediates the well-known (Andrykowski *et al.*, 1985) relationship between anxiety and nausea.

Delayed as compared with acute nausea was more strongly related to adrenergic activity. Since the nausea intensity was similar during acute and delayed nausea, it is not likely that individual modulating factors are of less importance only because nausea is more severe during chemotherapy. Instead, data may indicate that factors associated with adrenergic activation are prognostic specifically for delayed nausea.

It has been demonstrated that exogenous administration of catecholamines results in improved conditionability in animals (Weinberger *et al.*, 1984). Thus, to the extent that delayed nausea in part reflects conditioned nausea even in the second cycle of chemotherapy (Morrow, 1982; Andrykowski *et al.*, 1985; Burish & Carey, 1986; Hursti *et al.*, 1992), the data of the present study may also indicate that noradrenaline facilitates conditioning of nausea. We did not assess to what extent delayed nausea reflected conditioned nausea, and at present this must remain speculation.

We conclude that individual differences in delayed nausea after chemotherapy are influenced by neuroendocrine factors and linked both to the pituitary–adrenal cortical axis as indexed by cortisol (Fredrikson *et al.*, 1992; Hursti *et al.*, 1993) and to the sympathetic–adrenal medullary axis as reflected in noradrenaline. This may serve to tailor treatment strategies for patients likely to experience severe delayed emesis. It should support further study of neuroendocrine mechanisms involved in modulating chemotherapy emesis and particularly the possible role of the area postrema and the locus coeruleus and their interaction.

This study was supported by grants from The King Gustav V Jubilee Foundation and the Swedish Cancer Society. We are indebted to Kristina Bertilsson for technical assistance.

**References**

ANDREWS, P.L.R. & DAVIS, C.J. (1993). The mechanism of emesis induced by anti-cancer therapies. In *Emesis in Anti-cancer Therapy, Mechanisms and Treatment*, Andrews, P.L.R. & Sanger, G.J. (eds) pp. 113–161. Chapman & Hall: London.

ANDREWS, P.L.R., RAPEPORT, W.G. & SANGER, G.J. (1988). Neuropharmacology of emesis induced by anti-cancer therapy. *Trends Pharmaceut. Sci.*, **191**, 334–341.

ANDRYKOWSKI, M.A., REDD, W.H. & HATFIELD, A.K. (1985). Development of anticipatory nausea: a prospective analysis. *J. Consult. Clin. Psychol.*, **53**, 447–454.

BELESLIN, D.B. (1992). Neurotransmitter receptor subtypes related to vomiting. In *Mechanisms and Control of Emesis*, Colloque INSERM Vol. 223, Bianci, A.L., Grélot, L., Miller, A.D. & King, G.L. (eds) pp. 11–18. John Libbey Eurotext: London.

BELESLIN, D.B. & STRBAC, M. (1987. Noradrenaline-induced emesis: alpha-2 adrenoceptor mediation in the area postrema. *Neuropharmacology*, **26**, 1157–1165.

BORISON, H.L. (1974). Area postrema: chemoreceptor trigger zone for vomiting is that all? *Life Sci.*, **14**, 1807–1817.

BORISON, H.L. (1981). Phylogenic and neurologic aspects of the vomiting process. *J. Clin. Pharmacol.*, **21**, 23–29.

BORISON, H.L. (1984). History and status of the area postrema. *FASEB J.*, **43**, 2937–2940.

BORISON, H.L. (1989). Area postrema: chemoreceptor circumventricular organ of the medulla oblongata. *Prog. Neurobiol.*, **32**, 351–390.

BURISH, T.G. & CAREY, M.P. (1986). Conditioned aversive responses in cancer chemotherapy patients: theoretical and developmental analysis. *J. Consult. Clin. Psychol.*, **54**, 593–600.

DURANT, J.R. (1984). The problem of nausea and vomiting in modern cancer chemotherapy. *CA*, **34**, 2–6.

ELAM, M., SVENSSON, T.H. & THOREN, P. (1986). Locus coeruleus neurons and sympathetic nerves: activation by visceral afferents. *Brain Res.*, **375**, 117–125.

FREDRIKSON, M., HURSTI, T., FÜRST, C.-J., STEINECK, G., BÖRJESON, S., WIKBLOM, M. & PETERSON, C. (1992). Nausea in cancer chemotherapy is inversely related to urinary cortisol excretion. *Br. J. Cancer*, **65**, 779–780.

HARRIS, J.G. (1978). Nausea, vomiting and cancer treatment. *CA*, **28**, 194–201.

HJEMDAHL, P., LARSSON, P.T., BRADLEY, T., ÅKERSTEDT, T., ANDERZÉN, I., SIGURDSSON, K., GILLBERG, M. & LUNDBERG, U. (1989). Catecholamine measurements in urine by high-performance liquid chromatography with amperometric detection – comparison with an autoanalyser fluorescence method. *J. Chromatogr.*, **494**, 53–66.

HURSTI, T., FREDRIKSON, M., BÖRJESSON, S., FÜRST, C.-J., PETERSON, C. & STEINECK, G. (1992). Association between personality characteristics and the prevalence and extinction of conditioned nausea after chemotherapy. *J. Psychosoc. Oncol.*, **10**, 59–77.

HURSTI, T., FREDRIKSON, M., STEINECK, G., BÖRJESSON, S., FÜRST, C.-J. & PETERSON, C. (1993). Endogenous cortisol exerts antiemetic effect similar to that of exogenous corticosteroids. *Br. J. Cancer*, **68**, 112–114.

JENKINS, L.C. & LAHAY, D. (1971). Central mechanisms of vomiting related to catecholamines response: anaesthetic implication. *Can. Anaesth. Soc. J.*, **18**, 434–441.

JOHANSSON, S., STEINECK, G., HURSTI, T., FÜRST, C.J., FREDRIKSON, M. & PETERSON, C. (1992). Care of men with testicular cancer – interviews with relapse-free patients in Stockholm. *Cancer Nurs.*, **15**, 54–60.

LAZLO, J. & LUCAS, Jr, V.S. (1981). Emesis as a critical problem in chemotherapy. *N. Engl. J. Med.*, **305**, 948–949.

LESLIE, R.A. & REYNOLDS, D.J.M. (1993). Neurotransmitters and receptors in emetic pathway. In *Emesis in Anti-cancer Therapy, Mechanisms and Treatment*, Andrews, P.L.R. & Sanger, G.J. (eds) pp. 91–112. Chapman & Hall: London.

MORROW, G.R. (1982). Prevalence and correlates of anticipatory nausea and vomiting in chemotherapy patients. *J. Natl. Cancer Inst.*, **68**, 585–588.

RIGGIN, R.M. & KISSINGER, P.T. (1977). Determination of catecholamines in urine by reverse-phase liquid chromatography with electrochemical detection. *Anal. Chem.*, **49**, 2109–2111.

SAGAR, S. (1991). The current role of anti-emetic drugs in oncology: a recent revolution in patient symptom control. *Cancer Treat. Rev.*, **18**, 95–135.

SAMARDZIC, R. & BELESLIN, D.B. (1989). Neurochemical mechanisms in area postrema and emesis. *Jugoslovenska Physiologica et Pharmacologica Acta*, **25** (Suppl. 8), 133–153.

SMITH, D.B., NEWLANDS, E.S., SPRUYT, O.W., BEGENT, R.H.J., MELLOR, B. & BAGSHOWE, K.D. (1990). Ondansetron (GR38032F) plus dexamethasone: effective anti-emetic prophylaxis for patients receiving cytotoxic chemotherapy. *Br. J. Cancer*, **61**, 323–324.

SVENSSON, T.H. (1987). Peripheral, autonomic regulation of locus coeruleus noradrenergic neurons in brain: putative implications for psychiatry and psychopharmacology. *Psychopharmacology*, **92**, 1–7.

WEINBERGER, N.M., GOLD, P.E. & STERNBERG, D.B. (1984). Epinephrine enables Pavlovian fear conditioning under anesthesia. *Science*, **223**, 605–607.